# High Rates of Hepatitis C Virus Reinfection and Spontaneous Clearance of Reinfection in People Who Inject Drugs: A Prospective Cohort Study

**DOI:** 10.1371/journal.pone.0080216

**Published:** 2013-11-07

**Authors:** Rachel Sacks-Davis, Campbell K. Aitken, Peter Higgs, Tim Spelman, Alisa E. Pedrana, Scott Bowden, Mandvi Bharadwaj, Usha K. Nivarthi, Vijayaprakash Suppiah, Jacob George, Jason Grebely, Heidi E. Drummer, Margaret Hellard

**Affiliations:** 1 Centre for Population Health, Burnet Institute, Melbourne, Victoria, Australia; 2 Department of Epidemiology and Preventive Medicine, Monash University, Melbourne, Victoria, Australia; 3 Centre for Excellence into Injecting Drug Use, Burnet Institute, Melbourne, Victoria, Australia; 4 National Drug Research Institute, Curtin University, Melbourne, Victoria, Australia; 5 Victorian Infectious Diseases Reference Laboratory, Melbourne, Victoria, Australia; 6 Department of Microbiology and Immunology, University of Melbourne, Melbourne, Victoria, Australia; 7 Storr Liver Unit, Westmead Millennium Institute and Westmead Hospital, University of Sydney, Sydney, New South Wales, Australia; 8 School of Pharmacy, University of South Australia, Adelaide, South Australia, Australia; 9 Kirby Institute, University of New South Wales, Sydney, New South Wales, Australia; 10 Centre for Biomedical Research, Burnet Institute, Melbourne, Victoria, Australia; 11 Department of Microbiology, Monash University, Melbourne, Victoria, Australia; University of North Carolina School of Medicine, United States of America

## Abstract

Hepatitis C virus reinfection and spontaneous clearance of reinfection were examined in a highly characterised cohort of 188 people who inject drugs over a five-year period. Nine *confirmed* reinfections and 17 *possible* reinfections were identified (*confirmed* reinfections were those genetically distinct from the previous infection and *possible* reinfections were used to define instances where genetic differences between infections could not be assessed due to lack of availability of hepatitis C virus sequence data). The incidence of confirmed reinfection was 28.8 per 100 person-years (PY), 95%CI: 15.0-55.4; the combined incidence of confirmed and possible reinfection was 24.6 per 100 PY (95%CI: 16.8-36.1). The hazard of hepatitis C reinfection was approximately double that of primary hepatitis C infection; it did not reach statistical significance in confirmed reinfections alone (hazard ratio [HR]: 2.45, 95%CI: 0.87-6.86, p=0.089), but did in confirmed and possible hepatitis C reinfections combined (HR: 1.93, 95%CI: 1.01-3.69, p=0.047) and after adjustment for the number of recent injecting partners and duration of injecting. In multivariable analysis, shorter duration of injection (HR: 0.91; 95%CI: 0.83-0.98; p=0.019) and multiple recent injecting partners (HR: 3.12; 95%CI: 1.08-9.00, p=0.035) were independent predictors of possible and confirmed reinfection. Time to spontaneous clearance was shorter in confirmed reinfection (HR: 5.34, 95%CI: 1.67-17.03, p=0.005) and confirmed and possible reinfection (HR: 3.10, 95%CI: 1.10-8.76, p-value=0.033) than primary infection. Nonetheless, 50% of confirmed reinfections and 41% of confirmed or possible reinfections did not spontaneously clear.

**Conclusions:** Hepatitis C reinfection and spontaneous clearance of hepatitis C reinfection were observed at high rates, suggesting partial acquired natural immunity to hepatitis C virus. Public health campaigns about the risks of hepatitis C reinfection are required.

## Introduction

Hepatitis C virus (HCV) infects an estimated 170 million people worldwide and is a significant cause of morbidity and mortality [1,2]. In developed countries, HCV predominantly infects people who inject drugs (PWID) [3,4]. Spontaneous clearance of HCV occurs in 20-40% of primary infections [5]. However, HCV reinfection following spontaneous clearance of primary HCV infection raises concerns that natural immunity may be short-lived and/or have limited breadth, with implications for vaccine development [6] and public health programs.

Results from HCV reinfection studies in PWID have been contradictory [7,8], with some studies reporting very high rates of reinfection [9-14], and others reporting much lower rates [15-18]. In part these variations may be attributed to methodological limitations, including variations in test interval (where studies with lengthy test intervals do not observe short spontaneously clearing reinfections) [19], and classification of viral recurrence as reinfections without confirmation that the viraemic periods were genetically distinct [20]. None of these studies have been able to analyse predictors of reinfection, either because of insufficient numbers of reinfection events or lack of behavioural data. The aim of this study was to examine HCV reinfection and spontaneous clearance of reinfection among a well-characterized cohort of PWID in Melbourne, Australia (Networks II).

## Materials and Methods

### Ethics Statement

Participation was voluntary, written informed consent was obtained, and participants were offered pre- and post-test counseling. Ethical approval was obtained from the Victorian Department of Health Human Research Ethics Committee (project 02/05).

### Study design

Between 2005 and 2006, PWID who had injected in the previous six months were recruited from major street drug markets located across metropolitan Melbourne using modified snowball sampling [9]. All participants were bled and interviewed once using a structured questionnaire and selected participants were asked to participate in follow-up if they met one of the following criteria indicating risk of HCV infection:

- aged ≤25 years;
- duration of injecting less than four years;
- tested negative for HCV antibodies (anti-HCV); or
- tested HCV RNA negative.

Participants who were not selected for follow-up were recruited as injecting partners of the primary participants as part of a larger social network study, and were not included in this analysis. Follow-up (including blood sampling and structured interviews) was undertaken from 2005-2010 at locations convenient for participants. 

### Laboratory testing

Blood samples were screened for anti-HCV by a third-generation enzyme immunoassay (Abbott Laboratories, Chicago, Ill); anti-HCV positive specimens were retested by Murex anti-HCV version 4.0 (Murex Biotech, Kyalami, South Africa) for confirmation. Irrespective of anti-HCV status, samples were tested for HCV RNA by the COBAS AMPLICOR HCV test version 2.0 (Roche Molecular Systems, Branchburg, NJ; lower limit of detection - 50 IU/mL) at every visit. 

HCV RNA positive blood samples were genotyped by a reverse-phase hybridization line probe assay (Versant HCV Genotype Assay, Bayer, Tarrytown, NY) [21]. For molecular studies, amplification was performed using a nested in-house PCR with primers specific to the core region [22] and sequencing was performed on the PCR product using ABI PRISM^TM^ Dye Terminator Cycle Sequencing Ready Reaction Kit (PE Applied Biosystems, Foster City, CA). Methods for detecting other blood-borne viruses have been described elsewhere [23].

### Study definitions

#### Primary HCV infection

A participant’s first ever HCV infection was defined as their ‘primary’ HCV infection, with subsequent infections termed ‘reinfections’. Participants testing anti-HCV negative, HCV RNA positive at study entry were classified as having evidence of recent primary HCV infection. Participants testing anti-HCV negative and HCV RNA negative at study entry were considered at risk of primary infection. Those who subsequently became HCV RNA positive were defined as having a primary HCV infection. If participants were anti-HCV negative at the first HCV RNA positive test, the date of primary infection was defined as four weeks prior to the date of first HCV RNA positive test [24-26]. If participants were anti-HCV positive at the first HCV RNA positive test, the date of primary infection was defined as the midpoint between the date of the first HCV RNA positive test and the most recent HCV RNA negative test.

#### Intermittent viraemic events

Participants who entered the study anti-HCV positive, those with evidence of recent primary infection at baseline (anti-HCV negative but HCV RNA positive at study entry), and those with primary infection during the study were examined for intermittent viraemic events – defined as one or more HCV RNA negative tests followed by an HCV RNA positive test after primary HCV infection. 

#### Confirmed HCV reinfection

Intermittent viraemic events accompanied by the appearance of genetically distinct HCV (evaluated by analysis of HCV core region – details below) were defined as *confirmed HCV reinfections*. The date of confirmed reinfection was defined as the midpoint between the first HCV RNA positive test indicating reinfection and the previous HCV RNA negative test.

#### HCV intercalation

Intermittent viraemic events that were not accompanied by the appearance of genetically distinct HCV (evaluated by analysis of HCV core region – details below) were classified as HCV intercalations.

#### Possible HCV reinfection

Possible HCV reinfections were used to define instances where HCV sequence data was not available for either the participant’s first or second infection so genetic differences between infections could not be assessed. In these cases, events were classified as *possible HCV reinfections* if at least two consecutive HCV RNA negative tests (at least four weeks apart to confirm clearance) separated tests with detectable viremia. The date of possible reinfection was defined as the midpoint between the first HCV RNA positive test indicating reinfection and the previous HCV RNA negative test.

#### Risk periods for confirmed and possible HCV reinfection

**Figure 1:**
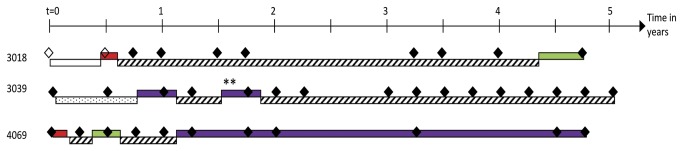
Classification of reinfections and risk periods: three illustrative examples. Diamonds indicate blood tests (empty diamonds denote anti-HCV negative bleeds and black diamonds denote anti-HCV positive bleeds). When participants are HCV RNA positive, viral genotype is indicated by colour (green: genotype 1; red: genotype 3; purple: genotype 6) . Change in viral subtype is indicated by a double asterisk. Periods at risk of infection are illustrated with patterned bars. Patterns indicate the type of infection for which the participant is at risk (no pattern: primary HCV infection; diagonal lines: confirmed HCV reinfection; dots: possible HCV reinfection. Participant 3018 is classified as at risk of primary HCV infection at study entry. Their second bleed is anti-HCV negative, HCV RNA positive indicating primary HCV infection, which is spontaneously cleared. Since viraemia was detected and sequenced at their primary infection, from spontaneous clearance they are classified as at risk of confirmed reinfection. In their final bleed, they have evidence of confirmed reinfection. Participant 3039 has one risk period for possible reinfection, a possible reinfection followed by a risk period for confirmed reinfection, a confirmed reinfection and another risk period for confirmed reinfection. Participant 4069 has two risk periods for confirmed reinfection and two confirmed reinfections.

Participants who had spontaneously cleared an infection during the study period that could be sequenced (and therefore could be compared to a subsequent infection to assess whether these were genetically distinct) were considered at risk of *confirmed* reinfection. Participants who had spontaneously cleared an infection that could not be sequenced, or had evidence of previous spontaneous clearance at baseline (anti-HCV positive and two consecutive HCV RNA negative tests at least four weeks apart), were considered at risk of possible reinfection (Figure 1). Risk periods began at study entry if the participant was initially uninfected, otherwise from time of spontaneous clearance. For participants who became infected during the study period and then cleared that infection, time at risk resumed from spontaneous clearance.

#### Spontaneous clearance

Spontaneous clearance was defined as two consecutive HCV RNA negative tests with at least four weeks between successive negative tests (in practice this interval was always ≥10 weeks). In addition, participants with one negative HCV RNA test followed by a positive HCV RNA test that was genetically distinct from the previous infection were defined as having spontaneous clearance followed by *confirmed* HCV reinfection. The date of spontaneous clearance was defined as the midpoint between the first HCV RNA negative test after an infection episode and the previous HCV RNA positive test. Participants with only one undetectable HCV RNA as their last measurement were included, but were not considered to have achieved spontaneous HCV clearance. However, because these participants may have achieved spontaneous clearance, a sensitivity analysis was undertaken with these participants excluded (Table S1).

#### Viral sequencing and definition of genetically distinct virus

All HCV RNA positive bleeds underwent viral sequencing (HCV core region, 331 nucleotides, methods described above). Viral sequences were compared pairwise, and the maximum composite likelihood distances were calculated. The mean distance between viral sequences taken from different participants but with the same genotype and subtype was 0.035 (standard deviation, 0.013). When consecutive sequences from the same participant were compared, 69.7% of consecutive sequences were identical (distance=0) and 90% had distance <0.010. Participants were defined as having a genetically distinct virus if they changed genotype or subtype or had two consecutive sequences with maximum composite likelihood sequence distance greater than 0.039 (three standard deviations of the distribution of pairwise differences from viral sequences from different participants with the same genotype and subtype). This approach was adapted from that used by Pham and colleagues [14]. 

### Statistical analyses

Baseline characteristics of participants with and without at least one follow-up visit, and of anti-HCV negative and anti-HCV positive participants, were compared using chi-squared tests (categorical variables) and Kruskal-Wallis equality-of-populations rank tests (continuous variables). To assess whether time to infection and time to clearance were different in primary infection compared to reinfection, and to identify predictors of reinfection separate to primary infection, gap-time unrestricted proportional hazards regression was used (appropriate for the analysis of predictors of time-to-event outcomes where participants can contribute multiple events to the analysis [27]). Interactions between infection type (primary infection vs reinfection) and predictors of infection were assessed. All analyses were undertaken for confirmed reinfections only and for confirmed and possible reinfections. 

Participants were included in the time-to-infection models if they were at risk of primary infection or reinfection. It was hypothesised that injecting duration [3], injecting frequency [9,28,29], recent receptive needle-sharing [30], and injecting with two or more people in the past three months [31] would be associated with HCV infection; selection of predictors for the final adjusted model was based on *a priori* hypotheses, strength of association in the unadjusted analyses, and guided by the rule of thumb that approximately ten events are required per additional predictor. Decisions regarding whether to model predictors as continuous or categorical variables are discussed in Appendix S1. Schoenfeld residuals were used to evaluate the proportional hazards assumption.

Participants were included in the time-to-clearance models if they became infected (primary or reinfection) during the study period. For these models, the date when first at risk was defined as the date of infection. The primary aim of the statistical models was to compare time to primary infection and reinfection and time to spontaneous clearance in primary infection and reinfection. Insufficient clearance events prohibited adjustment for confounding factors in the spontaneous clearance models. In all analyses, p<0.05 was considered significant. All statistical analyses were performed using Stata Version 11 (StataCorp LP, College Station, Texas).

## Results

### Participant characteristics

The study included 252 participants, 188 (75%) of whom returned for at least one follow-up and were included in this analysis. There was no statistically significant difference in age (median of 24 in both groups, p=0.053), gender (36% vs. 31% female, p=0.476), or ethnicity (78% vs. 89% of European descent, p=0.064) between participants with at least one follow-up and those without. 

Of the 188 participants with at least one follow-up, 70 (37%) were anti-HCV negative and 118 (63%) were anti-HCV positive at study entry. Anti-HCV negative participants and anti-HCV positive participants had similar socio-demographic characteristics (age, gender, ethnicity, housing, and drug most injected) at study entry (Table S2). However, anti-HCV negative participants who had been injecting for fewer years (median: 6 vs. 8 years, p=0.004), were less likely to report having ever engaged in receptive needle sharing (53% vs. 73%, p=0.006), were less likely to have been incarcerated (16% vs. 50%, p<0.001), and were less likely to have ever received drug treatment (64% vs. 95%, p<0.001, Table S2). Only two participants were HIV-infected, and these had persistent HCV-coinfection so were ineligible for the analyses described below. HBV infection was also uncommon; two anti-HCV negative participants and seven anti-HCV positive participants were infected with HBV at study entry. 

### Primary infection

Of the 70 anti-HCV negative participants at study entry, seven were HCV RNA positive and were therefore classified as having recent HCV primary infection. Of the remaining 63 participants, 19 subsequently became infected with HCV (148 person-years [PY], incidence rate: 12.8 per 100 PY, 95%CI: 7.7-20.0). Socio-demographic and behavioural characteristics of participants at risk of acquiring primary HCV infection and reinfection are presented in Table 1.

**Table 1:** Socio-demographic and behavioural characteristics of participants at risk of acquiring HCV infection^**a**^.

	Primary infection		Possible reinfection		Confirmed reinfection	
	At risk	Infected	At risk	Infected	At risk	Infected
Number of participants	63^b,c^	19^b,d^	40^c^	17^d^	19^c^	9^d^
Median age (IQR) - years	24 (22-27)	24 (22-27)	26 (23-33)	23 (21-29)	25 (21-29)	24 (20-29)
Gender						
- Female	22 (35)	4 (21)	14 (35)	7 (41)	7 (37)	2 (22)
- Male	41 (65)	15 (79)	26 (65)	10 (59)	12 (63)	7 (78)
Ethnicity						
- Of European descent	52 (83)	13 (68)	35 (88)	14 (82)	15 (79)	7 (78)
- Other	11 (17)	6 (32)	5 (13)	3 (18)	4 (21)	2 (22)
Median duration of injection (IQR) - years	7 (3-10)	5 (1-10)	9 (8-15)	9 (5-12)	10 (5-11)	6 (5-10)
Median number of injections in the past month (IQR)	20 (9-40)	30 (12-56)	40 (20-61)	48 (18-110)	27 (13-61)	15 (13-30)
Number of injecting partners in the past three months^e^						
- 0-1	8 (13)	2 (11)	7 (18)	2 (13)	1 (7)	0 (0)
- 2+	55 (87)	17 (89)	32 (82)	14 (88)	14 (93)	7 (100)
Receptive needle sharing in the past three months^e^						
- No	49 (78)	14 (74)	33 (83)	13 (76)	11 (61)	6 (67)
- Yes	14 (22)	5 (26)	7 (18)	4 (24)	7 (39)	3 (33)
Opiate substitution therapy in the past three months^e^						
- No	37 (59)	10 (53)	25 (63)	13 (76)	9 (50)	5 (56)
- Yes	26 (41)	9 (47)	15 (38)	4 (24)	9 (50)	4 (44)
Accommodation^e^						
- Unstable (homeless, boarding)	17 (27)	5 (26)	9 (23)	4 (24)	4 (22)	3 (33)
- Stable (own home, renting, living with parents)	46 (73)	14 (74)	31 (78)	13 (76)	14 (78)	6 (67)
Main drug injected in the past three months^e^						
**- Heroin**	37 (59)	10 (53)	28 (72)	12 (75)	12 (67)	5 (56)
- Other	26 (41)	9 (47)	11 (28)	4 (25)	6 (33)	4 (44)

^a^ All data are number (column %) unless otherwise specified. Participants who were at risk of primary and reinfection, or possible and confirmed reinfection at different points of the study are included in multiple columns.

^b^ The seven participants with evidence of recent primary HCV infection at study entry (anti-HCV negative, HCV RNA positive) were excluded because the period at risk of primary HCV infection was 0.

^c^ Time dependent quantities are defined at the time at which the participant was first defined as being at risk of the relevant infection type.

^d^ Time dependent quantities are defined at the time of infection.

^e^ Numbers do not add to total in some columns due to a small amount of missing data.

### Reinfection

**Figure 2:**
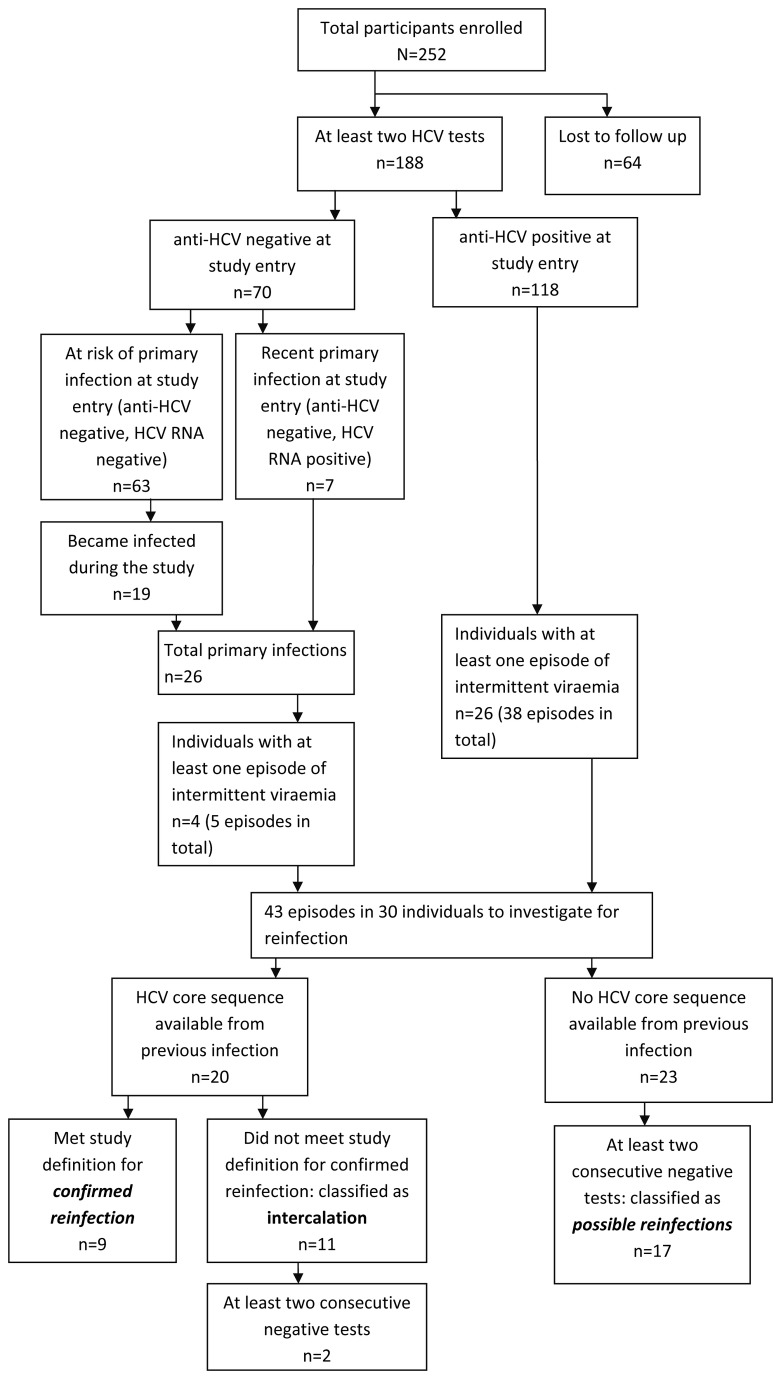
Flowchart of participant recruitment and identification of hepatitis C virus reinfections.

**Figure 3:**
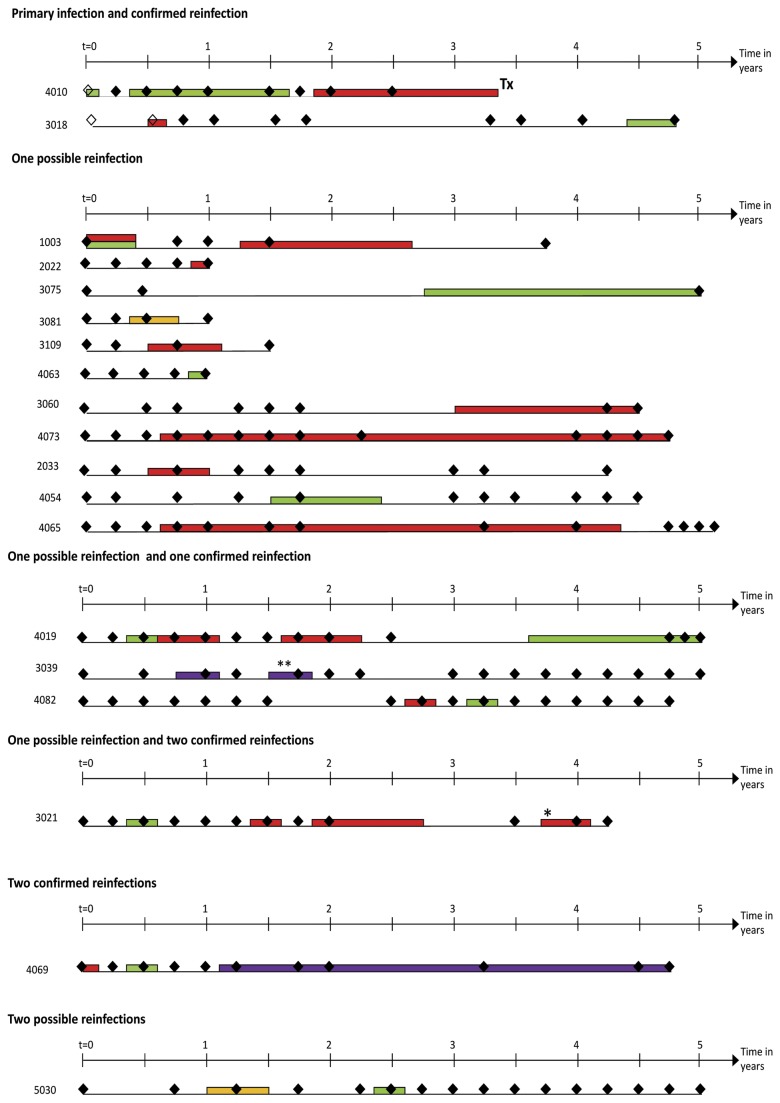
Hepatitis C reinfection timelines. Diamonds indicate blood tests (empty diamonds denote anti-HCV negative bleeds and black diamonds denote anti-HCV positive bleeds). When participants are HCV RNA positive, viral genotype is indicated by colour (green: genotype 1; red: genotype 3; purple: genotype 6; yellow: genotype could not be determined). Change in viral sequence is indicated by an asterisk (maximum composite likelihood difference between two consecutive sequences is greater than or equal to 0.04 but no change in viral subtype) or a double asterisk (change in viral subtype). Additional notes: participant 1003’s initial g1a infection could not be sequenced, participants 3018 and 5030’s initial infections could neither be genotyped nor sequenced.

Nine reinfections were confirmed in seven individuals (PY: 31, incidence rate: 28.8 per 100 PY, 95%CI: 15.0-55.4, Figures 2 & 3). These included seven reinfections with a different genotype, one with a different subtype, and one with a virus from the same genotype and subtype. The hazard of confirmed HCV reinfection was approximately double that of primary HCV infection but this did not reach statistical significance (hazard ratio [HR]: 2.45, 95%CI: 0.87-6.86, p=0.089; Table 2). 

**Table 2:** Gap-time unrestricted proportional hazards analysis of factors associated with time to HCV infection^**a**^.

	Confirmed reinfection only		Possible and confirmed reinfection			
	Univariable HR (95% CI)^b^	p-value	Univariable HR (95% CI)^b^	p-value	Multivariable HR (95% CI)^b^,^c^	p-value
Type of infection						
**- Primary infection**	1.00		1.00		1.00	
**- Reinfection**	2.45 (0.87-6.86)	0.089	**1.93 (1.01-3.69)**	**0.047**	**2.66 (1.26-5.62)**	**0.010**
Age (years)	0.97 (0.90-1.04)	0.426	0.96 (0.91-1.01)	0.099		
Gender						
**- Female**	0.56 (0.23-1.36)	0.200	0.79 (0.41-1.51)	0.468		
**- Male**	1.00		1.00			
Duration of injection - years	0.93 (0.85-1.02)	0.139	0.94 (0.88-1.00)	0.051	**0.91 (0.85-0.97)**	**0.007**
Frequency of injecting in the past month						
**- Less than daily**	1.00		1.00			
**- Daily or more**	1.87 (0.90-3.93)	0.096	1.47 (0.82-2.66)	0.199		
Number of injecting partners in the past three months						
**- 0-1**	1.00		1.00		1.00	
**- 2+**	2.52 (0.83-7.70)	0.104	**2.56 (1.08-6.03)**	**0.032**	**2.85 (1.21-6.70)**	**0.016**
**- Missing^d^**	1.41 (0.36-5.56)	0.622	1.30 (0.38-4.48)	0.667	1.64 (0.47-5.75)	0.437
Receptive needle sharing in the past three months						
**- No**	1.00		1.00			
**- Yes**	1.38 (0.57-3.39)	0.465	1.21 (0.63-2.32)	0.566		

^a^ There were no statistically significant interactions between infection type and the other predictors.

^b^ Controlled for infection number, as required by the gap-time unrestricted proportional hazards regression methodology

^c^ Test of proportional hazards: p=0.484

^d^ Small number of responses not collected due to a technical error.

Seventeen possible reinfection events occurred in 16 individuals (Figure 3). The overall incidence rate of possible and confirmed reinfection was 24.6 per 100 PY (PY: 106, 95%CI: 16.8-36.1). The hazard of confirmed and possible HCV reinfection was approximately double that of primary HCV infection and the difference was statistically significant (HR: 1.93, 95%CI: 1.01-3.69, p=0.047, Table 2), remaining similar after adjustment for the number of recent injecting partners and duration of injecting (adjusted HR: 2.66, 95%CI: 1.26-5.62, p=0.010, Table 2).

In univariable analyses, younger age and shorter duration of injection were statistically significant predictors of possible and confirmed reinfection. For each one year increase in age and duration of injection, the hazard of infection decreased by approximately 6% and 9% respectively (Table 3). Multiple injecting partners in the past three months tended to be associated with an approximately threefold increase in the hazard of possible and confirmed reinfection but this did not reach statistical significance (p=0.066, Table 3). Point estimates for the HRs of confirmed reinfection were similar for all three predictors but nine events gave insufficient power to detect a statistically significant effect (Table 3). In multivariable analysis, shorter duration of injection (HR: 0.91; 95%CI: 0.83-0.98; p=0.019) and multiple recent injecting partners (HR: 3.12; 95%CI: 1.08-9.00, p=0.035) were independent predictors of possible and confirmed reinfection (Table 3). There were no statistically significant differences in predictors of possible and confirmed reinfection compared to primary infection. 

**Table 3:** Gap-time unrestricted proportional hazards analysis of factors associated with time to HCV primary infection and reinfection.

	Primary infection only		Confirmed reinfection only		Possible and confirmed reinfection			
	Univariable HR (95% CI)	p-value	Univariable HR (95% CI)^a^	p-value	Univariable HR (95% CI)^a^	p-value	Multivariable HR (95% CI)^a^,^b^	p-value
Age (years)	0.97 (0.90-1.06)	0.527	0.97 (0.86-1.09)	0.600	**0.94 (0.88-1.00)**	**0.044**		
Gender								
- Female	1.73 (0.55-5.44)	0.351	1.94 (0.46-8.12)	0.363	1.06 (0.48-2.35)	0.889		
- Male	1.00		1.00		1.00			
Duration of injection - years	0.92 (0.81-1.04)	0.190	0.95 (0.80-1.12)	0.517	**0.91 (0.83-0.99)**	**0.032**	**0.91 (0.83-0.98)**	**0.019**
Frequency of injecting in the past month								
- Less than daily	1.00		1.00		1.00			
- Daily or more	1.66 (0.63-4.38)	0.304	1.24 (0.31-5.01)	0.760	0.97 (0.44-2.16)	0.948		
Number of injecting partners in the past three months								
- 0-1	1.00		1.00		1.00		1.00	
- 2+	2.13 (0.57-7.92)	0.261	3.19 (0.28-36.14)	0.348	2.75 (0.94-8.06)	0.066	**3.12 (1.08-9.00)**	**0.035**
- Missing^c^	1.14 (0.23-5.68)	0.877	1.06 (0.05-24.06)	0.969	1.21 (0.17-8.50)	0.848	1.70 (0.23-12.34)	0.598
Receptive needle sharing in the past three months								
- No	1.00		1.00		1.00			
- Yes	2.07 (0.80-5.33)	0.132	0.44 (0.05-3.70)	0.451	0.80 (0.34-1.90)	0.617		

^a^ Controlled for infection number, as required by the gap-time unrestricted proportional hazards regression methodology.

^b^ Test of proportional hazards: p=0.999

^c^ Small number of responses not collected due to a technical error.

Study retention characteristics were similar for primary infection, confirmed reinfection and possible reinfection. The median (IQR) years of follow-up from the time at risk of infection was four (1-5), three (1-5), and three (2-4), for primary infection, possible reinfection and confirmed reinfection, respectively. The median (IQR) number of tests during this time was six (4-9), eight (4-12), and seven (5-9), for primary infection, possible reinfection and confirmed reinfection respectively; tests were typically four (3-5), three (3,4), and three (3,4) months apart, respectively (Table S3). 

### Multiple reinfection

Six participants had multiple possible and/or confirmed reinfections. Five of these were anti-HCV positive, HCV RNA negative at study entry so their first episode of intermittent viremia could not be examined for confirmed reinfection. Three of these participants had one possible reinfection followed by one confirmed reinfection, one had one possible reinfection followed by two confirmed reinfections, and the fifth had two possible reinfections (viremia from the first possible reinfection could not be sequenced so the second reinfection could not be confirmed). The sixth participant was anti-HCV positive, HCV RNA positive at study entry and subsequently had two confirmed reinfections (Figure 3). 

The incidence of first and second confirmed reinfection, respectively, was 27.0 per 100 PY (7 cases, 26 PY, 95%CI: 10.9-55.7) and 37.5 per 100 PY (2 cases, 5 PY, 95%CI: 4.5-135.4). The incidence of first, second and third confirmed and/or possible reinfection, respectively, was 21.9 per 100 PY (19 cases, 87 PY, 95%CI: 13.2-34.2), 54.1 per 100 PY (6 cases, 11 PY, 95%CI: 19.9-117.8), and 13.0 per 100 PY (1 case, 8 PY, 95%CI: 0.3-72.5). 

### Intercalation

Eleven participants had HCV intermittent viraemia that was accompanied by the reappearance of very similar virus (determined by HCV core sequencing), and were classified as having HCV intercalation. Two of these were following at least two HCV RNA negative tests.

### Spontaneous Clearance

Of the 26 primary HCV infections, 24 had at least two HCV RNA tests after the estimated date of infection. Of these, six (25%) resulted in spontaneous clearance. In comparison, 50% of confirmed reinfections and 59% of confirmed or possible reinfections resulted in spontaneous clearance (Table 4). Time to spontaneous clearance was shorter in confirmed reinfection (HR: 5.34, 95%CI: 1.67-17.03, p=0.005) and confirmed or possible reinfection (HR: 3.10, 95%CI: 1.10-8.76, p-value=0.033) than primary infection. These results were not sensitive to the inclusion or exclusion of infections with only one undetectable HCV RNA as their last measurement (Table S1). Study retention characteristics after HCV infection were similar for primary infection, possible reinfection and confirmed reinfection (Table S3).

**Table 4:** Gap-time unrestricted proportional hazards analysis of infection type and time to HCV spontaneous clearance.

Type of infection	Number	Number with follow-up (%)	Clearances (%)	HR^a^ (95% CI)	p-value
- Primary infection	26	24 (92)	6 (25)	1.00	
- Reinfection (confirmed only)	9	8 (89)	4 (50)	**5.34 (1.67-17.03)^b^**	**0.005**
- Reinfection (possible and confirmed)	26	22 (85)	13 (59)	**3.10 (1.10-8.76)^c^**	**0.033**

^a^ Controlled for infection number, as required by the gap-time unrestricted proportional hazards regression methodology

^b^ Test of proportional hazards: p=0.405

^c^ Test of proportional hazards: p=0.832

## Discussion

We studied the pattern of HCV transmission and clearance in a highly characterised cohort of 188 PWID with frequent resampling over a five year period. The hazard of HCV reinfection was approximately double that of primary infection. Furthermore, some participants were reinfected more than once, illustrating the complexity of HCV natural history. Spontaneous clearance occurred at a higher rate in reinfection than primary infection; however, unlike in previous studies that found elevated rates of spontaneous clearance in reinfection compared to overall clearance of primary infection, in this study a substantial proportion of reinfections did not clear (approximately half). Both the elevated rate of spontaneous clearance in reinfection and the persistence of a considerable proportion of reinfections have implications for our understanding of acquired natural immunity to HCV. The persistence of a large proportion of reinfections also highlights the need for public health campaigns to educate PWID about the ongoing risk of infection after spontaneous HCV clearance, and potentially after successful antiviral treatment. Finally this study was uniquely positioned to investigate the predictors of reinfection, finding that shorter duration of injecting and number of recent injecting partners were independent predictors of possible or confirmed reinfection. 

The hazard of HCV reinfection was approximately double that of primary infection. Previous HCV reinfection studies have reported mixed results, some with higher and others with lower reinfection rates relative to primary infection rates [7-15,20]. Mathematical modelling has suggested that much of this variation is attributable to differences in study test intervals, and studies with lengthy test intervals miss spontaneously clearing reinfections that fall between study visits [19].A study with one-month test interval reported an average duration of reinfection of less than two months [12]. Therefore, while our three-month test interval was shorter than many previous reinfection studies, the reinfection rate observed in this study probably underestimates the true reinfection rate in this cohort; this highlights that reinfection is likely to be extremely common in this group. 

Reinfection rates may be overestimated by misclassifying fluctuations in viral load as reinfection [20]. However, this study found a very high rate of confirmed reinfection using a rigorous definition that required reinfections to be genetically distinct from previous infections. A similar rate of HCV reinfection was found using a less rigorous definition. Further differences between studies in relative rates of HCV reinfection to primary infection may be due to differences in genotype distribution (reinfection with heterologous genotype may occur at a higher rate than reinfection with homologous genotypes), and differences in injecting risk behaviours between participants at risk of primary infection or reinfection. Notably, participants at risk of reinfection have already been infected with HCV, and may therefore be more likely to engage in risk behaviours. In this study, the rate of possible and confirmed reinfection remained significantly higher than the rate of primary infection even after adjusting for the number of recent injecting partners; however, it is possible that this was due to residual confounding between injecting risk behaviours and infection type. 

Not only was the rate of reinfection very high, but some participants experienced multiple reinfections in the five year study period. Two participants had evidence of two confirmed reinfections, and six had evidence of two or more possible or confirmed reinfections. Small numbers of participants with multiple reinfections have been reported previously [12], and in combination these reports illustrate that the process of HCV infection, spontaneous clearance and reinfection is very dynamic, and suggests that either immune memory protecting PWID from future infection if they continue to expose themselves to HCV is limited, or cross-reactive immune responses are insufficient. 

While reinfection rates were high, spontaneous clearance was more common in reinfection than primary infection and time to spontaneous clearance was approximately four times faster in reinfection than in primary infection, similar to results reported by Osburn and colleagues [12]. This elevated rate of spontaneous clearance in reinfection is heartening in its implication that partial acquired immunity against future *persistent* HCV infection is likely [32]. Nonetheless, by definition all participants with reinfection previously cleared a primary HCV infection, so over time persistent infection is becoming more common in the reinfection group. In addition, unlike previous studies with elevated rates of spontaneous clearance in reinfection compared to primary infection, in this study a considerable proportion of reinfections did not spontaneously clear (approximately half). This reinforces the fact that acquired natural immunity to HCV is likely to be partial. It also highlights the potential health risks associated with HCV reinfection, raising the importance of public health initiatives to educate PWID about the risks of HCV reinfection following spontaneous HCV clearance. Moreover, although HCV reinfection following successful antiviral treatment has occurred infrequently in the past [16,33-38], with the advent of new highly effective treatments [39] and potential increases in the number of PWID being treated, reinfection following antiviral treatment may become common and will require close study.

Previous reinfection studies have been limited in their ability to identify factors associated with reinfection due to small numbers of reinfection events or lack of data on injecting risk factors, making this study uniquely positioned to investigate predictors of reinfection. Consistent with previously identified predictors of primary HCV infection, shorter duration of injecting [3] and more than one recent injecting partner [31,40] were found to be independent predictors of HCV reinfection after controlling for the infection number (that is, first infection observed in the study, second infection, etc). 

### Limitations

It is possible that the number of confirmed HCV reinfections was underestimated for the following reasons. Genetic sequencing was limited to the HCV core region, which is relatively conserved so some changes in viral sequence may not have been detected. Furthermore, repeat reinfections from same injecting partner would have been classified as intercalations rather than reinfections if there was no detectable change in viral sequence. 

Study participants were selected using modified snowball sampling and may not be representative of all PWID. Further, while the attrition rate was similar to other community-based studies of PWID [31,41] and socio-demographic characteristics were similar amongst those retained and those lost to follow-up, it is possible that attrition led to selection bias. Nonetheless, given that few PWID receive medical care for HCV, study participants may be more representative of PWID than those attending health services for care [42,43]. In addition, the participants of this study were recruited from street illicit drug markets and report high risk behaviour. While it is possible that they are more risky than the “average” PWID, documenting the natural history of HCV infection in this high risk group is important for informing prevention of HCV. 

Finally, similar to the other HCV reinfection studies conducted to date [9-16,18,20], the number of reinfection and spontaneous clearance events observed was small, limiting the potential to adjust for confounding factors in the regression analyses. In particular, this meant that there was very limited power to analyse predictors of confirmed reinfection separate to possible reinfection, and it was not possible to conduct a multivariable analysis of time to spontaneous clearance.

## Conclusion

This study found high rates of confirmed and possible reinfection, including some participants with more than one reinfection within a five-year follow-up period. Shorter duration of injecting and having multiple recent injecting partners were independent predictors of possible or confirmed reinfection. The rate of spontaneous clearance was high in both confirmed and possible reinfection relative to primary infection, suggesting that previous infection with HCV confers some immunity against future persistent infection. However, many reinfections did not clear, demonstrating that immunity is incomplete. These findings highlight the complexity of HCV natural history in PWID, and that the risk of reinfection should be communicated to PWID as part of public health programs. 

## Supporting Information

Appendix S1 **Modelling predictors as continuous vs. categorical variables.**
(DOCX)

Table S1 **Sensitivity analysis for gap-time unrestricted proportional hazards analysis of infection type and time to HCV spontaneous clearance.**
(DOCX)

Table S2 **Baseline socio-demographic and behavioural characteristics by HCV antibody status.**
(DOCX)

Table S3 **Study retention characteristics relevant to time to infection and time to spontaneous clearance analyses.**
(DOCX)
