# Supplementary material for: High Rates of Hepatitis C Virus Reinfection and Spontaneous Clearance of Reinfection in People Who Inject Drugs: A Prospective Cohort Study
Source: PLoS One. 2013 Nov 7;8(11):e80216. doi: 10.1371/journal.pone.0080216 (PMC3820644; doi:10.1371/journal.pone.0080216)
Supplement: Appendix S1 — Modelling predictors as continuous vs. categorical variables. (DOCX) [file pone.0080216.s001.docx]

Appendix S1: Modelling predictors as continuous vs. categorical variables

Age, duration of injecting, frequency of injecting and number of injecting partners were collected as continuous variables. It was hypothesised that having multiple injecting partners in the three months prior to interview would be a risk factor for HCV infection similar to the effect of having multiple sexual partners on the risk of STI, so number of injecting partners was treated as a categorical variable (0-1 partners vs. 2+ partners). The remaining three variables were explored as continuous and categorical variables (with cut-offs for categories close to the median values). Because of the small numbers of events being modeled, continuous variables were preferred as long as the assumption of proportional increase in the log hazard with increase in the predictor variable was reasonable. This was assessed by modeling the variables with more than two categories to ensure that the hazard ratios tended to increase or decrease monotonically, assessing the effects of extreme values by removing them, and scrutinizing the Martingale residuals. On the basis of these investigations, it was decided to model frequency of injecting a categorical variable (less than daily vs. daily) and age and duration of injecting as continuous variables.
