# Supplementary material for: High Rates of Hepatitis C Virus Reinfection and Spontaneous Clearance of Reinfection in People Who Inject Drugs: A Prospective Cohort Study
Source: PLoS One. 2013 Nov 7;8(11):e80216. doi: 10.1371/journal.pone.0080216 (PMC3820644; doi:10.1371/journal.pone.0080216)
Supplement: Table S1 — Sensitivity analysis for gap-time unrestricted proportional hazards analysis of infection type and time to HCV spontaneous clearance. (DOCX) [file pone.0080216.s002.docx]

**Table S1:** **Sensitivity analysis for gap-time unrestricted proportional hazards analysis of infection type and time to HCV spontaneous clearance**

| Type of infection | Inclusion of participants with only one HCV RNA negative as their last measurements^a^ | Number | Number with follow-up (%) | Clearances (%) | HR (95% CI)^b^ | p-value |
| --- | --- | --- | --- | --- | --- | --- |
| - Primary infection | Included | 26 | 24 (92) | 6 (25) | 1.00 |  |
| - Primary infection | Excluded | 26 | 23 (88) | 6 (26) | 1.00 |  |
| - Reinfection (confirmed only) | Included | 9 | 8 (89) | 4 (50) | **5.34 (1.67-17.03)** | **0.005** |
| - Reinfection (confirmed only) | Excluded | 9 | 7 (78) | 4 (57) | **5.06 (1.59-16.15)** | **0.006** |
| - Reinfection (confirmed or possible) | Included | 26 | 22 (85) | 13 (59) | **3.10 (1.10-8.76)** | **0.033** |
| - Reinfection (confirmed or possible) | Excluded | 26 | 18 (69) | 13 (72) | **3.87 (1.37-10.96)** | **0.011** |

^a^In the main analysis (Table 4), participants with only one undetectable HCV RNA as their last measurement were included, but not considered to have achieved spontaneous HCV clearance and were censored at the last HCV RNA test. However, because these participants may have achieved spontaneous clearance, a sensitivity analysis was also undertaken with these participants excluded.

^b^Controlled for infection number, as required by the gap-time unrestricted proportional hazards regression methodology.
