# Supplementary material for: High Rates of Hepatitis C Virus Reinfection and Spontaneous Clearance of Reinfection in People Who Inject Drugs: A Prospective Cohort Study
Source: PLoS One. 2013 Nov 7;8(11):e80216. doi: 10.1371/journal.pone.0080216 (PMC3820644; doi:10.1371/journal.pone.0080216)
Supplement: Table S2 — Baseline socio-demographic and behavioural characteristics by HCV antibody status. (DOCX) [file pone.0080216.s003.docx]

**Table S2: Baseline socio-demographic and behavioural characteristics by HCV antibody status^a^**

| HCV antibody status | Negative | Positive | p-value |
| --- | --- | --- | --- |
| Number of participants | 70 | 118 |  |
| Median age (IQR) | 24 (21-27) | 24 (21-26) | 0.434 |
| Gender |  |  |  |
| - Male | 44 (63) | 75 (64) | 0.864 |
| - Female | 26 (37) | 42 (36) |  |
| Ethnicity |  |  |  |
| - Of European descent | 57 (81) | 88 (75) | 0.324 |
| - Other | 13 (19) | 29 (25) |  |
| Median duration of injection (IQR) - years | 6 (3-9) | 8 (5-11) | 0.004 |
| Accommodation |  |  |  |
| - Unstable (homeless or boarding house) | 20 (29) | 38 (32) | 0.619 |
| - Stable (own home, renting or living with parents) | 49 (71) | 79 (68) |  |
| Drug most injected |  |  |  |
| - Heroin | 43 (62) | 82 (70) | 0.276 |
| - Other | 26 (38) | 35 (30) |  |
| Receptive needle sharing ever |  |  |  |
| - Yes | 37 (53) | 85 (73) | 0.006 |
| - No | 33 (47) | 32 (27) |  |
| Incarcerated ever |  |  |  |
| - Yes | 11 (16) | 58 (50) | <0.001 |
| - No | 59 (84) | 59 (50) |  |
| Drug treatment ever^b^ |  |  |  |
| - Yes | 45 (64) | 111 (95) | <0.001 |
| - No | 25 (36) | 6 (5) |  |

^a^All data are number (column %) unless otherwise specified.

^b^Including opiate substitution therapy, counselling, and other forms of drug treatment.
