# Supplementary material for: High Rates of Hepatitis C Virus Reinfection and Spontaneous Clearance of Reinfection in People Who Inject Drugs: A Prospective Cohort Study
Source: PLoS One. 2013 Nov 7;8(11):e80216. doi: 10.1371/journal.pone.0080216 (PMC3820644; doi:10.1371/journal.pone.0080216)
Supplement: Table S3 — Study retention characteristics relevant to time to infection and time to spontaneous clearance analyses. (DOCX) [file pone.0080216.s004.docx]

**Table S3: Study retention characteristics relevant to time to infection and time to spontaneous clearance analyses**

| Study retention from | Infection type | Years of follow-up | Number of follow-up tests | Test interval (months) |
| --- | --- | --- | --- | --- |
| Time at risk of infection | Primary infection | 4 (1-5) | 6 (4-9) | 4 (3-5) |
| Time at risk of infection | Possible reinfection | 3 (1-5) | 8 (4-12) | 3 (3-4) |
| Time at risk of infection | Confirmed reinfection | 3 (2-4) | 7 (5-9) | 3 (3-4) |
| Estimated date of infection | Primary infection | 2 (1-4) | 5 (3-7) | 3 (3-4) |
| Estimated date of infection | Possible reinfection | 3 (2-4) | 10 (2-11) | 3 (3-4) |
| Estimated date of infection | Confirmed reinfection | 3 (1-4) | 7 (5-9) | 3 (3-4) |

* All data are median (IQR).
